# Metabolomic Characterization of Baby Spinach Phenolics Transformation During Gastrointestinal Digestion and Microbiome-Mediated Metabolism

**DOI:** 10.3390/foods15111893

**Published:** 2026-05-27

**Authors:** Akhtar Ali, Leqi Cui

**Affiliations:** Department of Health, Nutrition, and Food Sciences, Florida State University, Tallahassee, FL 32306, USA; aali4@fsu.edu

**Keywords:** baby spinach, storage impact, metabolomic outputs, digestibility, bioaccessibility, gut health benefits

## Abstract

Gastrointestinal digestion and colonic fermentation determine phenolic transformation and reciprocal microbiome modulation, influencing the generation of gut-derived metabolites associated with epithelial integrity, inflammatory regulation, and metabolic homeostasis. Baby spinach phenolics possess antioxidant and microbiome-modulating potential; however, their functional efficacy is constrained by storage-induced degradation and limited gastrointestinal bioaccessibility. This study investigated phenolic transformation in fresh and stored baby spinach (4 °C and 25 °C) during simulated gastrointestinal digestion and subsequent colonic fermentation. Standardized in vitro digestion revealed limited phenolic bioaccessibility (10–15%), with storage at 25 °C accelerating oxidative degradation and reducing antioxidant capacity. Storage at 25 °C reduced TPC from approximately 465 to 265 µg GAE/g and decreased antioxidant activity by nearly 30%, whereas refrigerated storage (4 °C) better preserved phenolic stability and antioxidant capacity throughout the storage period. LC–MS/MS–based untargeted metabolomics characterized digestion-driven structural remodeling and identified diverse colonic metabolites generated during human fecal fermentation. Despite storage-induced alterations in precursor phenolics, 16S rRNA sequencing demonstrated microbiome relative microbial stability, with fermentation time exerting a stronger influence on community assembly than storage conditions. Microbial metabolism produced shared downstream metabolites, particularly phenylpropionic and flavonoid-derived intermediates. These results suggest that storage modifies phenolic availability during digestion, while gut microbial metabolism sustains the production of functionally relevant metabolites.

## 1. Introduction

Green leafy vegetables are rich sources of structurally diverse phytochemicals, particularly phenolic compounds, which contribute to antioxidant, anti-inflammatory, and cardiometabolic health benefits [1,2,3,4]. Spinach (*Spinacia oleracea*) is widely recognized as a functional food due to its high content of flavonoids, phenolic acids, and carotenoids. Among these, flavonol glycosides—especially derivatives of spinacetin, patuletin, and quercetin—are characteristic of spinach and play a central role in its bioactive profile [5]. However, these compounds are highly sensitive to postharvest conditions, and their stability is strongly influenced by storage temperature and duration. Previous work from our group applied LC-ESI-QTOF-MS/MS-based metabolomics and chemometric analysis to characterize the impact of storage conditions on phenolic stability and antioxidant activity in baby spinach. That study demonstrated that refrigeration preserves key flavonoids such as patuletin and spinacetin derivatives, whereas storage at elevated temperatures accelerates oxidative degradation and structural remodelling [5]. However, the investigation was limited to compositional changes during storage and did not extend to the fate of these compounds during gastrointestinal digestion or their subsequent interaction with the gut microbiome.

Beyond postharvest stability, the nutritional and functional relevance of spinach phenolics depends on their bioaccessibility and microbial transformation in the gastrointestinal tract. A substantial proportion of phenolic compounds are not absorbed in the small intestine and instead reach the colon, where they undergo extensive microbial metabolism [6]. This process generates a diverse array of low-molecular-weight metabolites, including phenylpropionic, phenylacetic, and hydroxybenzoic acid derivatives, which may exert systemic biological effects [7]. Furthermore, emerging evidence suggests that fermentation time may exert a stronger influence on microbial community assembly and metabolite production than upstream storage-induced differences in phenolic composition, reflecting the dynamic and adaptive nature of gut microbial metabolism [8]. Thus, the health benefits of spinach are not solely determined by its native phenolic composition but also by the metabolic capacity of the gut microbiome.

Emerging evidence indicates that dietary patterns rich in fruits and leafy vegetables promote enrichment of saccharolytic and short-chain fatty acid (SCFA)-producing microbial taxa, including *Bacteroides*, *Prevotella*, *Bifidobacterium*, and members of the *Ruminococcaceae*. These taxa possess specialized enzymatic systems capable of degrading complex plant polysaccharides and phenolic conjugates. Microbial metabolism of flavonoids and hydroxycinnamic acids involves sequential reactions such as deglycosylation, dehydroxylation, demethylation, and aromatic ring cleavage, ultimately funneling structurally diverse compounds into common metabolic intermediates [9,10]. These transformations are tightly coupled with microbial cross-feeding networks that support SCFA production and ecosystem stability. Recent studies further demonstrate that food processing and postharvest handling can substantially influence the bioaccessibility, structural stability, and microbial transformation of dietary phenolics. Storage-associated modifications in plant matrices may alter the availability of phenolic substrates reaching the colon, thereby influencing gut microbial metabolism and the generation of bioactive gut-derived metabolites. These interactions highlight the importance of integrating food processing variables with microbiome-centered nutritional research.

Despite growing interest in food–microbiome interactions, limited studies have integrated postharvest storage effects with gastrointestinal digestion and colonic fermentation. In particular, it remains unclear how storage-induced modifications in spinach phenolics influence their bioaccessibility and subsequent microbial metabolism. We hypothesized that: (i) postharvest storage temperature and duration significantly influence phenolic stability, structural integrity, and release during gastrointestinal digestion; (ii) digestion-driven transformations alter the diversity and composition of phenolic substrates reaching the colon; and (iii) despite storage-induced modifications in precursor phenolics, gut microbial communities exhibit functional convergence by transforming diverse substrates into shared catabolic intermediates during colonic fermentation. Furthermore, because microbial fermentation is inherently dynamic and time-dependent, (iv) we hypothesized that fermentation duration would exert a stronger influence on microbial community assembly and metabolite production than storage conditions alone. Addressing the gap and testing these hypotheses is critical for understanding how pre-consumption factors shape the functional output of plant-based diets. Therefore, the present study integrates postharvest storage, simulated gastrointestinal digestion, and in vitro fecal fermentation to evaluate the dynamic transformation of spinach phenolics. By linking upstream storage-induced compositional changes to downstream microbial metabolism, this work provides a comprehensive framework for understanding how food-processing variables influence gut-derived bioactive metabolites. LC–ESI–QTOF–MS/MS-based untargeted metabolomics was used to characterize phenolic transformations and gut-derived metabolites, while 16S rRNA gene sequencing examined microbial community dynamics. By integrating storage chemistry, digestive bioaccessibility, and microbiome metabolism, this study offers a systemic understanding of how postharvest handling influences the functional fate of spinach phenolics from ingestion to colonic transformation. It advances current knowledge by linking food processing variables to gut microbial metabolomic outcomes and informs strategies to improve storage methods whilst safeguarding microbiome-mediated functional potential.

## 2. Materials and Methods

### 2.1. Sample Collection and Materials Used in the Experiment

Baby spinach (Mykonos and SV2157 varieties) leaves were collected from Publix (transported by Fresh Express under refrigeration) and stored at refrigeration temperature (4 °C) or room temperature (25 °C) for 2–9 days. The storage experiment utilized two biological and three technical replicates. Fresh and stored spinach (270 g each) was chopped, stored at −80 °C, and freeze-dried for at least 72 h using a VisTis BenchTop K freezer dryer (SP Industries Inc., Warminster, PA, USA). Dried samples, each weighing 270 g, were ground using a Cuisinart spice and nut grinder. Biological replicates were combined to produce homogeneous samples for each storage interval [5]. Gallic acid monohydrate (398225), Folin & Ciocalteu’s phenol reagent (47641), sodium carbonate (222321), aluminium chloride hydrate (229393), sodium acetate trihydrate (S8625), quercetin (PHR1488), 2,2-diphenyl-1-picryl-hydrazyl (D9132), ethylenediaminetetraacetic acid (E6758), ferrous chloride (372870), ferrozine (P9762), Trolox (238813), formic acid 98–100% for LC-MS (5.33002), and acetonitrile hypergrade for LC-MS (1.00029) were purchased from Sigma Aldrich (St. Louis, MO, USA). HPLC-grade Ethanol (AC611050040) and Methanol (A452SK-4) were purchased from Thermo Fisher Scientific (Waltham, MA, USA), as also described in our study [5]. Moreover, pepsin, pancreatin, bile, peptone, yeast extract, NaHCO_3_, Tween 80, mucin, sodium cholate, sodium chenodeoxycholate, NaCl, KCl, (NH_4_)CO_3_, MgCl_2_(H_2_O)_6_, K_2_HPO_4_, KH_2_PO_4_, MgSO_4_, and CaCl_2_·2H_2_O, *L*-cysteine hydrochloride, hemin, and vitamin K1 were purchased from Sigma-Aldrich.

### 2.2. In Vitro Simulated Gastrointestinal Digestion

The INFOGEST 2.0 method of Brodkorb et al. [11] was employed in this study, with adjustments as needed. To investigate the impact of continuous digestion on the production of colonic metabolites, fresh spinach (raw) and stored spinach at 4 °C (denoted with C for cold) and 25 °C (denoted with R for room) for up to nine days (2, 4, 6, and 9), underwent simulated in vitro oral, gastric, and intestinal digestion, followed by in vitro colonic fermentation. The samples analyzed for digestion consisted of 1 g of each treated freeze-dried baby spinach, as described in Section 2.1 [5]. All samples and controls (without added sample) underwent continuous-stage gastrointestinal digestion and in vitro colonic fermentation in triplicate. The gastrointestinal digestion simulation was performed according to the INFOGEST 2.0 methodology with modifications [11,12]. Simulated salivary fluid (SSF), simulated gastric fluid (SGF), and simulated intestinal fluid (SIF) were prepared as previously described. One gram of each sample was mixed with 4 mL of water to form a simulated oral digestion mixture. To this, 4 mL of SSF, 25 μL of 0.3 M CaCl_2_ (H_2_O)_2_, and 975 μL of water were added to a 50 mL centrifuge tube, and the mixture was vortexed for 3 min at 37 °C in a Roto-Therm TM incubated rotator (Benchmark Scientific, Edison, NJ, USA). Controls without any samples were included. The oral-phase digest was mixed with 7.5 mL of simulated gastric fluid (SGF), 1.6 mL of porcine pepsin stock solution (25,000 U/mL), 5 μL of 0.3 mol/L CaCl_2_, and 675 μL of distilled water. The pH was lowered to approximately 3.0 using about 0.1 mL of 1 M HCl. The reaction tubes were then placed in a shaking incubator set to 37 °C and incubated for 2 h to allow gastric digestion. Following the gastric stage, the digesta was transitioned to the intestinal phase. The gastric mixture was supplemented with 12.5 mL of simulated intestinal fluid (SIF), 2.5 mL of freshly prepared pancreatin solution (800 U/mL), 1.25 mL of freshly prepared fed-state bile extract (40 mg/mL), 20 μL of 0.3 mol/L CaCl_2_, and 655 μL of distilled water. The pH was adjusted to approximately 7.0 by adding around 75 μL of 1 M NaOH. The samples were then incubated at 37 °C under continuous rotation for an additional 2 h. Upon completion of the intestinal digestion stage, samples were collected and stored at −80 °C until further analysis.

### 2.3. Preparation of Basal Media and Fecal Slurry

The basal culture medium was formulated according to the method described by Li et al. [13]. Briefly, the medium was prepared in one liter of distilled water containing 2 g peptone, 2 g yeast extract, 4 g sodium bicarbonate (NaHCO_3_), 2 mL Tween 80, 4 g mucin, 0.25 g sodium cholate, 0.25 g sodium chenodeoxycholate, 1 g sodium chloride (NaCl), 0.4 g dipotassium hydrogen phosphate (K_2_HPO_4_), 0.4 g potassium dihydrogen phosphate (KH_2_PO_4_), 0.1 g magnesium sulfate (MgSO_4_), and 0.1 g calcium chloride dihydrate (CaCl_2_·2H_2_O). The pH was adjusted to 7.0 at 25 °C using either 1 mol/L HCl or 1 mol/L NaOH. The prepared medium was then sterilized by autoclaving at 121 °C for 20 min. After sterilization, the medium was transferred into an anaerobic chamber for pre-reduction. It was subsequently enriched with filter-sterilized stock solutions to obtain final concentrations of 0.5 g/L L-cysteine hydrochloride, 5 mg/L hemin, and 10 µL/L vitamin K_1_.

Fresh fecal samples were obtained from three healthy male volunteers aged between 25 and 35 years within 30 min of defecation for slurry preparation. Fecal samples collected from three healthy adult male donors. Donors had not consumed antibiotics, probiotics, or specialized dietary supplements within three months prior to sample collection. Approximately 10 g of faecal material was suspended in three volumes of pre-reduced, nitrogen-flushed 0.1 M phosphate buffer (pH 7.0) and thoroughly homogenized by vortex mixing. The homogenate was passed through sterile muslin cloth to remove particulate debris. The resulting filtrate was diluted with phosphate-buffered saline (PBS) to prepare a 2% (*w*/*v*) inoculum. Aliquots of the prepared fecal slurry were dispensed into sterile 50 mL Falcon tubes and further portioned into Eppendorf tubes for use in experiments conducted on the same day. All handling procedures involving fecal material were carried out under aseptic conditions in a biosafety cabinet, in accordance with established laboratory guidelines. In this study, three individual biological and two technical replicates were used.

### 2.4. In Vitro Colonic Fermentation

The in vitro colonic fermentation procedure was conducted according to the methodology of Pérez-Burillo et al. [14]. The intestinal-phase mixture was thawed, heated to 37 °C, and then centrifuged at 4000 rpm for 15 min at 4 °C to isolate the precipitate for the colonic fermentation procedure. Pre-prepared fecal slurry and basal media were incubated in a digestion chamber at 37 °C under nitrogen gas and 5% carbon dioxide. Five millilitres of each fecal slurry and basal medium were added to the fermentation tubes containing 300 mg of intestinal-phase precipitate and 10 µL of intestinal supernatant. All capped tubes were thereafter positioned in a shaking incubator (New Brunswick Scientific, Edison, NJ, USA) set at 100 rpm and 37 °C for 48 h. Samples were obtained at four distinct time points (0, 12, 24, and 48 h) from various tubes during the colonic fermentation phase, rapidly frozen in liquid nitrogen, and stored at −80 °C for subsequent analysis.

### 2.5. Measurement of Total Phenolic Content and Antioxidant Activities

#### 2.5.1. Preparation of Sample Extracts

Baby spinach polyphenols were extracted using 80% acidified ethanol (0.1% formic acid) by mixing at 4 °C at 150 rpm for 16 h, followed by centrifugation at 4000 rpm and 4 °C for 20 min. The resultant supernatant was filtered through a 0.22 µm syringe filter and stored at −20 °C prior to analysis [5]. In vitro-digested samples obtained from the gastrointestinal phases were centrifuged at 4000 rpm and 4 °C for 20 min, yielding supernatant and precipitate for the assessment of total phenolic content (TPC), TFC, DPPH, and FICA. The digesta supernatants utilized for the TPC, TFC, DPPH, and FICA quantification were prepared by combining the in vitro digested supernatant with acidified ethanol (0.1% formic acid) in a 1:1 ratio (*v*/*v*) through vortexing for 30 min, followed by centrifugation at 4000 rpm and 4 °C for 20 min and filtered using a 0.45 µm syringe filter to yield the final supernatants for final analysis.

#### 2.5.2. Quantification of Phenolic Compounds in Baby Spinach

Total phenolic content (TPC) was quantified according to the method of Vallverdú-Queralt et al. [15], with minor modifications to accommodate microplate analysis. Briefly, 25 μL of phenolic extract was combined with 25 μL of 25% Folin–Ciocalteu reagent and 200 μL of Milli-Q water in a 96-well microplate. After incubation in the dark for 5 min, 25 μL of 10% (*v*/*v*) sodium carbonate solution was added to initiate chromophore development. The reaction mixture was further incubated for 60 min at room temperature prior to measuring absorbance at 765 nm using a BioTek Synergy H1 microplate reader (Agilent Technologies, Winooski, VT, USA). Quantification was performed using a gallic acid calibration curve (0–200 μg/mL), and results were expressed as mg gallic acid equivalents (GAE) per g of freeze-dried sample. The calibration curve equation was y = 0.0319x + 0.0043, with an R^2^ value of 0.9984.

Total flavonoid content (TFC) was determined using a modified aluminum chloride colourimetric assay adapted from Subbiah et al. [16]. An aliquot (80 μL) of extract was mixed with 80 μL of 2% AlCl_3_ solution and 120 μL of 50% (*w*/*v*) aqueous sodium acetate in a 96-well plate. Following incubation in the dark at 22 °C for 2.5 h, absorbance was recorded at 440 nm. Quantification was based on a quercetin standard curve (0–50 μg/mL in methanol), and TFC was expressed as mg quercetin equivalents (QE) per g of sample. The calibration curve equation was y = 0.0136x + 0.0631, with an R^2^ value of 0.999.

#### 2.5.3. Measurement of the Impact of Storage on the Antioxidant Potential of Baby Spinach

Radical-scavenging activity was evaluated using the DPPH assay following Ebrahimi et al. [17] with minor modifications. Briefly, 25 µL of phenolic extract was mixed with 275 µL of 0.1 mM DPPH solution and incubated in the dark at 22 °C for 30 min. Absorbance was recorded at 517 nm using a microplate spectrophotometer (Agilent Technologies, Winooski, VT, USA). Ethanol blanks and reagent controls were included to correct for background absorbance. All measurements were performed in triplicate. Quantification was based on a Trolox calibration curve (0–50 µg/mL; y = −0.0047x + 1.1174, R^2^ ≥ 0.995), and results were expressed as mg Trolox equivalents (TE) per g freeze-dried sample.

Ferrous ion chelating activity (FICA) was determined according to Ebrahimi et al. [18] with slight modifications. The reaction mixture consisted of 15 µL extract, 85 of µL distilled water, 50 µL of 2 mM FeCl_2_, and 50 µL of 5 mM ferrozine. After incubation at 25 °C for 10 min, absorbance was measured at 562 nm. Reagent blanks were used for baseline correction, and assays were conducted in triplicate. Quantification was performed using an EDTA standard curve (0–50 µg/mL; y = −0.0174x + 1.0996, R^2^ ≥ 0.997), and results were expressed as mg EDTA equivalents per g sample.

### 2.6. Extraction and Sequencing of 16S rRNA

Each replicate from all samples collected at various time points during the in vitro colonic fermentation was thawed and centrifuged at 10,000× *g* for 10 min to remove phenolic compounds that interfere with DNA extraction. Bacterial DNA was extracted using the DNeasy^®^ PowerSoil^®^ Pro Kit (QIAGEN GmbH, Hilden, Germany), and 16S rRNA sequencing was carried out by the Department of Biological Sciences at Florida State University, United States. The V4 region of the 16S rRNA gene was amplified by PCR using the 27F and 519R primers. PCR amplification was carried out under the following conditions: initial denaturation at 95 °C for 3 min, followed by 30 cycles of denaturation at 95 °C for 30 s, annealing at 55 °C for 30 s, and extension at 72 °C for 45 s, with a final extension at 72 °C for 5 min. Sequencing was performed on an Illumina MiSeq (San Diego, CA, USA) using a V3, 600-cycle kit (2 × 300 paired-end reads).

### 2.7. Individual Phenolic Compounds Assessment in Baby Spinach Using LC-ESI-QTOF-MS/MS

Phenolic metabolites were isolated and characterized following our previously established protocol Ali et al. [5]. Phytochemical profiling was performed using an Agilent LC–ESI–QTOF–MS/MS system (Accurate-Mass Q-TOF LC/MS) coupled to an Agilent 1200 HPLC series (Agilent Technologies, Santa Clara, CA, USA). Data acquisition and processing were conducted using MassHunter Workstation software (version B.06.00, Agilent Technologies). Untargeted phenolic analysis was carried out on preserved baby spinach extracts using an Agilent Technologies InfinityLab Poroshell 2.7 μm SB-C18 column (150 × 2.1 mm, 120 Å), protected by a UHPLC guard column (2.1 × 5.0 mm). A 1 μL aliquot of each extract was injected at a flow rate of 0.2 mL/min. The mobile phases consisted of (A) 0.1% formic acid in Milli-Q water and (B) 0.1% formic acid in acetonitrile, using the following gradient: 0–10 min (10–20% B), 10–20 min (20–25% B), 20–30 min (25–30% B), 30–40 min (30–45% B), 40–50 min (45–60% B), 50–65 min (60–90% B), 65–67 min (90–100% B), 67–68 min (100–10% B), and 68–70 min (10% B re-equilibration). Mass spectrometric parameters were set as follows: scan range 100–1000 *m*/*z*, nitrogen gas flow 9 L/min at 350 °C, capillary voltage 3500 V, nebulizer pressure 45 psi, and collision energies of 10, 20, and 40 eV for MS/MS fragmentation in positive mode. Metabolite identification was performed using MassHunter Qualitative Analysis (version B.06.00), the Agilent Personal Compounds Database and Library (PCDL), and other online databases. Metabolite annotation was performed using accurate mass, MS/MS fragmentation patterns, retention behavior, and database/spectral library matching. A pooled sample of all extractions was used for quality control, and all samples were conducted in triplicate.

### 2.8. Data Analysis

Sequence data were processed using QIIME2 (version 2025.4) [PMID: 31341288]. Raw reads were demultiplexed and quality-filtered (Q > 20) with the q2-demux plugin, followed by trimming and denoising using DADA2 [19]. Amplicon sequence variants (ASVs) were aligned with MAFFT [20]. Taxonomic classification was performed using a pre-trained naïve Bayes classifier implemented in scikit-learn, trained on the SILVA 138 database (99% sequence similarity). Alpha diversity was evaluated using the Chao1 (richness) and Shannon (richness and evenness) indices, with group comparisons assessed via the non-parametric Kruskal–Wallis test (α = 0.05). Beta diversity was evaluated using the Bray–Curtis dissimilarity metric, and community differences were visualized through principal coordinates analysis (PCoA). Statistical significance in community composition among groups was assessed using the PERMANOVA with 999 permutations. For univariate analyses, statistical significance was assessed using appropriate analysis of variance followed by post hoc comparisons, and multiple testing was controlled using false discovery rate (FDR) correction where applicable. PLS-DA models were validated using cross-validation and permutation testing. Furthermore, VIP scores were interpreted cautiously and used as screening indicators in combination with fold change, clustering behavior, and metabolite annotation confidence rather than as standalone evidence of biomarker significance. Although VIP > 1 is frequently used as a conventional threshold for selecting discriminatory metabolites, VIP thresholds are model- and dataset-dependent. Therefore, a more inclusive threshold (VIP > 0.7) was applied in this exploratory metabolomics study to retain metabolites that contributed moderately to downstream biological interpretation, alongside criteria for fold-change and statistical significance.

## 3. Results and Discussion

### 3.1. Impact of In Vitro Gastrointestinal Digestion on the Total Phenolic Content, Total Flavonoid Content, and Their Antioxidant Activities of Fresh and Stored Baby Spinach

**Figure 1:**
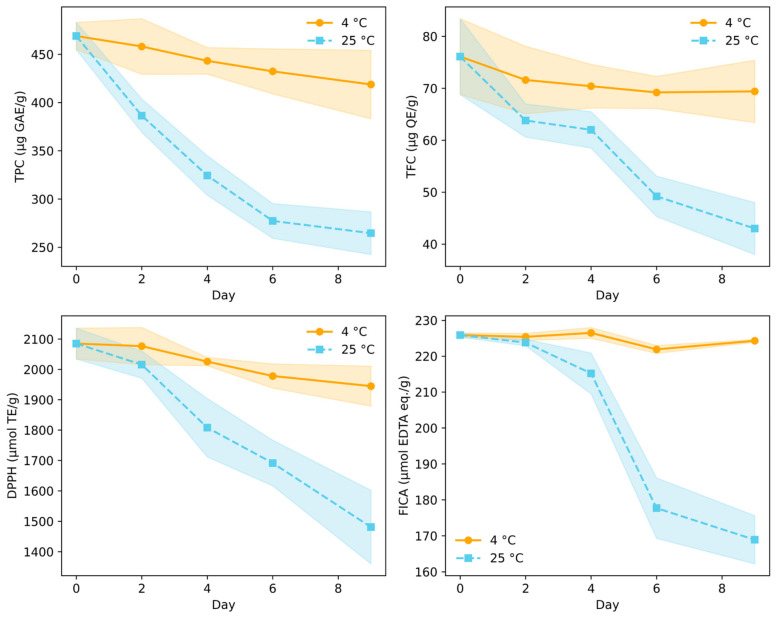
Effect of storage temperature on phenolic retention and antioxidant activity in digested baby spinach. Total phenolic content (TPC), total flavonoid content (TFC), DPPH radical scavenging activity, and ferrous ion chelating activity (FICA) were measured in raw (0 days) and stored spinach (4 °C and 25 °C; 2–9 days). Values are expressed as µg equivalents per gram of digested sample (GAE, QE, TE, and EDTA equivalents, respectively).

This study extends our previous metabolomics-based investigation of storage-induced phenolic changes in baby spinach [5] by integrating gastrointestinal digestion and microbial fermentation. While earlier work established the impact of storage temperature on phenolic stability, the present study evaluates how these compositional changes influence bioaccessibility and downstream microbial metabolism, providing a more comprehensive understanding of spinach-derived bioactive compounds. The impact of storage on the bioaccessibility of phenolic compounds in baby spinach was assessed using simulated oral, gastric, and intestinal digestion of samples stored at 4 °C and 25 °C for up to nine days (Figure 1).

Storage temperature and duration significantly influenced the fraction of phenolics that remained available after digestion. Total phenolic content (TPC) and total flavonoid content (TFC) in digested samples declined progressively with increasing storage time, with markedly greater reductions at 25 °C. After 6 and 9 days at room temperature, bioaccessible TPC and TFC were significantly lower than in refrigerated samples, indicating that elevated temperature accelerates pre-digestive degradation and reduces the pool of compounds released during digestion. In contrast, storage at 4 °C better preserved phenolic stability, resulting in comparatively higher bioaccessible fractions throughout the experimental period.

Regardless of storage conditions, gastrointestinal digestion further reduced the levels of measurable phenolics [21,22]. Only approximately 10–15% of the total phenolics and flavonoids were estimated to be bioaccessible after digestion, suggesting substantial degradation or transformation during the digestive phases [22]. The intestinal stage likely contributed most to these losses, as the alkaline pH promotes oxidation, hydrolysis, and structural rearrangements of flavonoids and phenolic acids [23,24]. Deglycosylation of flavonoid glycosides may generate unstable aglycones that are more susceptible to breakdown, while interactions with proteins, fiber, and digestive enzymes may reduce extractability as previously reported by Kotik et al. [25] and Slámová et al. [26]. Samples stored at 25 °C consistently exhibited lower post-digestive phenolic levels than those stored at 4 °C, confirming that improper storage compounds the destabilizing effects of gastrointestinal conditions.

Antioxidant activities measured by DPPH and FICA assays followed similar trends. DPPH scavenging activity declined from approximately 2080 to 1480 µg TE/g after prolonged storage at 25 °C, while FICA decreased from approximately 226 to 169 µg EDTA eq./g. The proportional decline in antioxidant capacity exceeded the decrease in total phenolics, suggesting that highly active compounds are preferentially degraded or structurally altered during digestion. Storage at 25 °C further intensified these losses, indicating cumulative effects of thermal degradation and digestive instability. These findings demonstrate that both storage conditions and digestive processes critically influence the functional availability of phenolic compounds, emphasizing the importance of refrigeration to preserve not only total phenolic content but also their bioaccessible antioxidant potential.

### 3.2. Impact of In Vitro Gastrointestinal Digestion on the Individual Phenolic Profile of Fresh and Stored Baby Spinach

**Figure 2:**
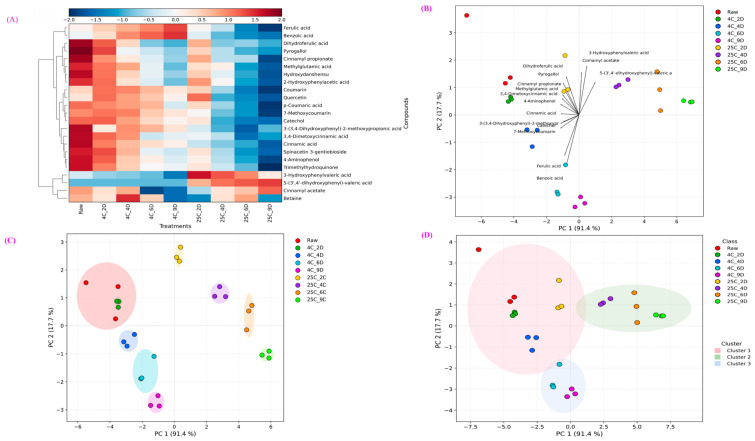
The changes in the profile of the digested phenolics of raw (0 days) and stored spinach (4 °C and 25 °C; 2–9 days). (**A**) Heatmap of individual phenolic compounds detected during in vitro digestion, (**B**) PCA biplot, (**C**) PLS-DA scores plot, and (**D**) K-means clustering illustrating treatment separation and storage-dependent trends in phenolic composition.

The results of the storage impact on individual phenolic compounds during in vitro digestion are presented in Figure 2.

Heatmap clustering (Figure 2A) reveals clear temperature-dependent alterations in metabolite levels. Fresh spinach (day 0) and samples stored at 4 °C for 2 and 4 days exhibited similar abundance patterns, indicating minimal compositional changes. In contrast, samples stored at 25 °C for 6 and 9 days displayed pronounced reductions in key phenolics, including quercetin, ferulic acid, benzoic acid, coumarin, and *p*-coumaric acid, confirming significant degradation under elevated temperature. Conversely, certain metabolites, such as cinnamyl acetate, 3-hydroxyphenylvaleric acid, and 5-(3′,4′-dihydroxyphenyl)-valeric acid, increased during prolonged storage at 25 °C, suggesting secondary metabolite formation via oxidative transformation or breakdown pathways. Refrigerated storage preserved or slightly enhanced the levels of benzoic acid, ferulic acid, *p*-coumaric acid, and quercetin, reinforcing the protective role of low temperature. These observations are consistent with our previous metabolomics study, which demonstrated that refrigeration preserves key flavonoids such as patuletin and spinacetin derivatives, whereas elevated temperatures accelerate their degradation (Ali et al. 2025) [5]. The present findings further indicate that these storage-induced transformations influence not only phenolic abundance but also the subsequent metabolic fate of these compounds during digestion and fermentation.

Multivariate analyses further supported these observations. The biplot (Figure 2B) and PLS-DA score plot (Figure 2C) demonstrated that fresh samples and those stored at 4 °C for short durations clustered closely together, indicating limited metabolic divergence. In contrast, samples stored at 25 °C for 4–9 days formed distinct clusters, with PC1 explaining 91.4% of the total variance, confirming that temperature is the primary driver of metabolomic variation. K-means clustering (Figure 2D) segregated samples into three groups: fresh and short-term storage (2 days), prolonged refrigerated storage (4–9 days at 4 °C), and prolonged ambient storage (4–9 days at 25 °C). The clear separation of the 25 °C cluster highlights accelerated phenolic degradation and transformation at room temperature. Collectively, these findings demonstrate that refrigeration maintains phenolic integrity and bioaccessible antioxidant potential during storage and subsequent digestion, whereas storage at 25 °C induces significant degradation and promotes the formation of secondary metabolites, particularly after 6 and 9 days.

Simulated gastrointestinal digestion markedly reduced total phenolic content and antioxidant activity in baby spinach, and these reductions were strongly influenced by prior storage conditions. Samples stored at 25 °C showed substantially lower bioaccessible phenolics and antioxidant activity than refrigerated samples, particularly after prolonged storage, indicating that elevated temperature accelerates oxidative degradation and compromises digestive stability. Only approximately 10–15% of total phenolics remained bioaccessible after digestion, suggesting extensive structural transformation during the gastrointestinal phases. The intestinal phase likely contributed most to these losses because alkaline pH, bile salts, and enzymatic hydrolysis promote oxidation, deglycosylation, and rearrangement of flavonoids and phenolic acids. Similar reductions in phenolic bioaccessibility during digestion have been reported in other plant matrices, including buritirana and sea buckthorn products. Nevertheless, low recovery of intact phenolics does not necessarily indicate complete functional loss, as many compounds reach the colon in modified or bound forms that remain available for microbial metabolism [22,27,28,29,30].

The digestive stability of spinach phenolics was strongly structure-dependent. Hydroxycinnamic acids such as ferulic and p-coumaric acids exhibited greater relative stability, whereas quercetin derivatives were more susceptible to alkaline degradation and oxidative cleavage, particularly after storage at 25 °C. Refrigerated storage better preserved flavonols and hydroxycinnamic acids, resulting in comparatively higher post-digestive levels. In addition to degradation, gastrointestinal digestion also promoted the release and transformation of previously bound phenolics. Several metabolites, including dihydroferulic acid, catechol, cinnamic acid derivatives, and phenylvaleric acids, appeared after digestion despite not being detected in undigested spinach, suggesting enzymatic release and oxidative or hydrolytic conversion during digestion. The disproportionate decline in antioxidant activity relative to total phenolics further indicates that highly redox-active structures were preferentially degraded under intestinal conditions [28,31,32,33,34,35,36].

Importantly, considerable amounts of phenolic compounds and their derivatives likely reached the colon, where they were transformed by microbes into low-molecular-weight metabolites with potential biological activity. During colonic fermentation, structurally diverse phenolic substrates converged toward shared microbial catabolic intermediates, particularly phenylpropionic and phenylacetic acid derivatives, supporting the concept of functional redundancy in microbial aromatic metabolism. These findings demonstrate that phenolic bioaccessibility in baby spinach is governed by the combined effects of storage-induced oxidative modification, intrinsic structural stability, matrix interactions, and gastrointestinal transformation pathways. Refrigeration mitigated pre-digestive oxidative stress and better-preserved phenolic integrity, whereas storage at 25 °C amplified digestive losses and altered downstream metabolite profiles. Overall, the results suggest that the functional value of spinach phenolics depends not only on their initial abundance but also on their stability during storage and digestion and their capacity to generate biologically relevant gut-derived metabolites [30,31,32].

### 3.3. Impact of Fresh and Stored Baby Spinach on the Gut Microbiome During In Vitro Colonic Fermentation

**Figure 3:**
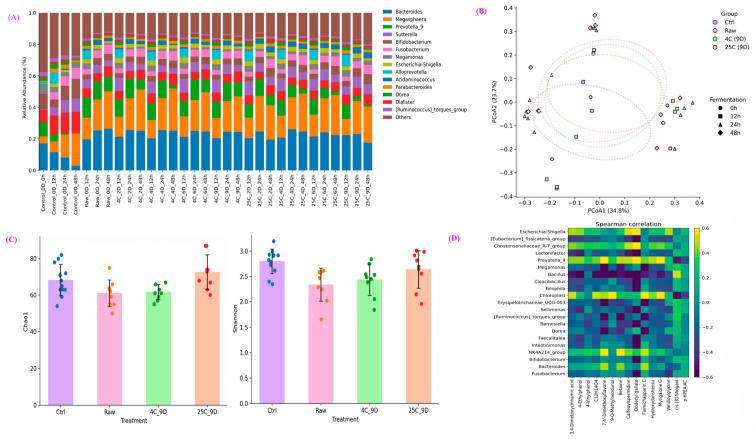
Microbial community structure and metabolite-microbe associations during the in vitro colonic fermentation of baby spinach. (**A**) Relative abundance of dominant bacterial genera across treatments as determined by 16S rRNA gene sequencing, displayed as stacked bar plots. (**B**) PCoA based on Bray–Curtis dissimilarity illustrating differences in community composition among treatments. (**C**) Alpha diversity metrics (Shannon and Chao1 indices) were used to compare microbial diversity across treatment groups. (**D**) Spearman correlation heatmap showing associations between abundant bacterial genera and high-variance metabolites.

Microbial community dynamics during in vitro colonic fermentation of fresh and stored baby spinach were characterized using 16S rRNA gene sequencing to evaluate taxonomic composition, diversity, and treatment-driven shifts in gut microbiota structure (Figure 3).

16S rRNA gene sequencing at the genus level demonstrated that microbial communities across all treatments were consistently dominated by *Bacteroides*, *Megasphaera*, *Prevotella*, and *Sutterella*, with additional contributions from *Bifidobacterium*, *Megamonas*, *Fusobacterium*, *Dialister*, *Dorea*, and members of the *[Ruminococcus]_torques_group* (Figure 3A). This compositional structure remained broadly conserved across treatments, including control fermentations and fermentations of raw and stored baby spinach, indicating overall stability in dominant gut microbial taxa during the in vitro fermentation process.

Alpha diversity analysis revealed moderate differences in microbial diversity among treatments (Figure 3B). Shannon diversity varied significantly between treatments (*p* < 0.05), indicating changes in microbial community evenness during fermentation. In contrast, Chao1 richness did not differ significantly among treatments (*p* > 0.05), suggesting that fermentation primarily influenced the relative abundance of taxa rather than overall taxonomic richness. Control samples generally exhibited slightly higher Shannon diversity compared with spinach treatments, whereas stored spinach samples showed modest reductions in diversity, consistent with selective enrichment of metabolically competitive fermentative taxa.

Bray–Curtis beta-diversity analysis was further conducted to explore the differences in microbial community composition among treatments (Figure 3C). Principal coordinate analysis (PCoA) showed partial separation between control and spinach fermentation samples, while raw spinach and stored spinach samples exhibited overlapping clustering patterns. PERMANOVA analysis confirmed that treatment significantly influenced microbial community structure (*F* ≈ 2.4, *p* = 0.001), explaining approximately 19% of the total variation in microbial composition. Pairwise PERMANOVA comparisons indicated that the strongest differences occurred between control and stored spinach treatments, whereas differences between raw and stored spinach samples were less pronounced. Cold storage (4 °C) of spinach for nine days resulted in minimal compositional divergence after fermentation, as reflected by the overlapping clustering of 4 °C and raw spinach samples in the PCoA ordination. Samples stored at 25 °C for nine days showed slightly greater dispersion, suggesting minor shifts in microbial composition; however, these changes did not substantially alter the dominance of core fermentative genera such as *Bacteroides* and *Megasphaera* [37].

The enrichment of *Bacteroides* during fermentation is notable because this genus is known to utilize carbohydrate-active enzyme systems that enable the degradation of complex plant polysaccharides, facilitating the fermentation of plant-derived dietary substrates. These metabolic capabilities likely support cross-feeding interactions within the microbial community during spinach fermentation.

Spearman correlation analysis further revealed associations between specific bacterial genera and metabolites detected in fermented spinach samples (Figure 3D). In particular, *Bacteroides* AND *Megasphaera* showed positive correlations with several fermentation-derived metabolites, suggesting coordinated microbial and metabolic responses during substrate fermentation. These relationships support the role of dominant fermentative taxa in shaping metabolite production during in vitro colonic fermentation.

Spinach and other leafy vegetables are rich in dietary fiber and phenolic compounds, including hydroxycinnamic acids and flavonols such as quercetin derivatives [2,5]. During digestion, a portion of these compounds reaches the colon, where they serve as substrates for microbial metabolism intestine modulators [38,39,40]. The selective fermentation of these plant-derived substrates promotes enrichment of saccharolytic and short-chain fatty acid-producing bacteria, including *Bacteroides*, *Prevotella*, and *Bifidobacterium*, reinforcing metabolic pathways associated with propionate and butyrate production [41,42]. Mechanistically, *Bacteroides* species hydrolyze complex plant glycans and phenolic conjugates via extensive polysaccharide utilization loci, liberating hydroxycinnamic acids and smaller phenolic intermediates [43,44]. These intermediates undergo microbial reduction and side-chain modification to phenylpropionic and phenylacetic acids, which can influence cross-feeding pathways and SCFA synthesis [44,45]. Overall, the results demonstrate that spinach fermentation alters microbial community composition primarily through shifts in relative abundance rather than major taxonomic restructuring. Importantly, the preservation of dominant fermentative taxa across raw and stored spinach treatments suggests that microbial fermentative functionality remains stable despite pre-fermentation storage conditions. These findings highlight the potential of spinach-derived substrates to sustain stable microbial fermentation processes even under variable storage conditions, thereby supporting their application in sustainable food systems and functional food development.

### 3.4. Impact of In Vitro Colonic Fermentation on Gut Metabolites Production During Reciprocal Interactions with the Baby Spinach Phenolics and the Gut Microbiome

**Figure 4:**
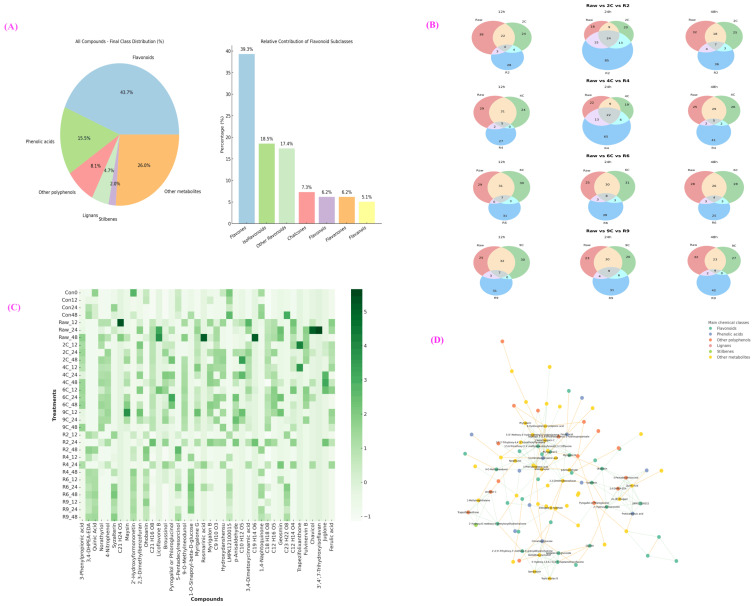
The phenolic metabolites after gut fermentation of raw and stored spinach (4 °C and 25 °C; 2–9 days). (**A**) Chemical class distribution of detected metabolites and relative abundance of flavonoid sub-classes. (**B**) Venn diagrams showing shared and unique metabolites across storage conditions in raw and stored spinach. (**C**) Heatmap of selected metabolites across treatments during 12, 24 and 48 h in vitro colonic fermentation. (**D**) Molecular network illustrating structural relationships among me-tabolite classes. In the Venn diagram, R represents 25 °C, while C represents 4 °C, with spinach stored for 2, 4, 6, and 9 days.

Beyond small-intestinal bioaccessibility, a substantial proportion of spinach phenolics reach the colon intact, where microbial metabolism generates structurally diverse secondary metabolites with potential systemic bioactivity [46]. In this study, reciprocal interactions between the gut microbiome and digested baby spinach resulted in the tentative identification of 400 gut-derived metabolites, including 178 flavonoids, 63 phenolic acids, 33 other phenols, 19 lignans, 8 stilbenes, and 108 additional metabolites (Figure 4A, Figure S1). 

Flavonoids constituted the largest class, particularly flavones and isoflavonoids, indicating that spinach-derived conjugates undergo extensive colonic remodeling rather than simple degradation. These findings highlight the metabolic versatility of the gut microbiota in transforming complex plant phenolics into structurally reconfigured bioactive compounds. The extensive diversity of detected metabolites indicates that spinach phenolics undergo progressive microbial remodeling rather than simple degradation. This convergence toward shared metabolic endpoints highlights the functional redundancy of microbial aromatic compound metabolism and supports the robustness of gut microbial ecosystems in processing structurally diverse dietary substrates. Venn distribution analysis (Figure 4B) demonstrated that storage conditions modulated the diversity of fermentation products. After 12 h of fermentation, raw spinach produced 38 unique metabolites, compared with 28 (25 °C, 2 days) and 24 (4 °C, 2 days). Interestingly, the highest number of unique metabolites was observed after 24 h of fermentation in samples stored for 4 days at 25 °C (R4), suggesting that moderate storage-induced phenolic transformation may increase microbial substrate diversity. Storage, extended storage at 25 °C, led to a decline in unique metabolites, consistent with oxidative degradation limiting downstream microbial conversion. In contrast, refrigerated storage preserved phenolic precursors and supported progressive increases in metabolite diversity across fermentation timepoints. The heatmap of top discriminant metabolites (VIP > 0.7) (Figure 4C) revealed substantially higher metabolite abundance in spinach-treated samples compared to fermentation controls, confirming that spinach-derived phenolics drive significant metabolic output. The molecular network (Figure 4D) highlighted dense structural clustering around quercetin, isorhamnetin, and related derivatives, suggesting these compounds act as central hubs in microbial biotransformation pathways. Mechanistically, flavonols such as quercetin undergo deglycosylation followed by ring fission, demethylation, and side-chain shortening, yielding phenylpropionic, phenylacetic, and hydroxybenzoic acid derivatives. Similarly, hydroxycinnamic acids (e.g., ferulic and *p*-coumaric acids) are reduced to dihydro derivatives and further metabolized through β-oxidative pathways [47]. These intermediates can integrate into cross-feeding networks, linking primary phenolic degraders with short-chain fatty acid (SCFA)-producing taxa [48]. These findings align with previous research on postharvest losses of leafy greens and enhance comprehension by linking storage-induced phytochemical alterations to gut fermentation outcomes. The combined effect on antioxidant defence and microbiota modulation highlights the nutritional significance of appropriate storage methods.

Although room-temperature storage accelerated phenolic breakdown and reduced antioxidant capacity, colonic fermentation converged toward overlapping classes of downstream metabolites across treatments. This convergence suggests that diverse flavonoids and hydroxycinnamic acids are converted into shared microbial catabolic endpoints—particularly phenylpropionic and phenylacetic acid derivatives—thereby demonstrating functional redundancy within microbial aromatic metabolism [49,50]. Reciprocal interactions between phenolics and the gut microbiome extend beyond substrate conversion. Phenolic compounds can modulate microbial gene expression, membrane stability, and redox balance, exerting selective pressures that suppress opportunistic taxa while favoring metabolically competitive fermenters [51,52]. In turn, microbial transformation enhances phenolic bioactivity by generating smaller, more absorbable metabolites with distinct physiological properties [53]. Thus, colonic fermentation represents a dynamic bidirectional interface: phenolics shape microbial ecology, and microbial metabolism reshapes phenolic structure.

Collectively, these findings demonstrate that postharvest storage modulates the qualitative diversity of phenolic substrates entering colonic fermentation, yet fermentation time remains the dominant determinant of gut metabolite production. Refrigeration preserves structural precursors and supports sustained metabolite diversity, whereas prolonged room-temperature storage narrows the range of substrates. Nevertheless, the persistence of major metabolite classes and the dense connectivity of molecular networks suggest relatively stable microbial phenolic metabolism under the experimental conditions. This integration of storage chemistry and colonic biotransformation underscores the importance of postharvest handling in shaping gut-derived bioactive metabolite profiles while reinforcing the robustness of microbiome-mediated metabolic conversion.

### 3.5. Proposed Pathway of Identified Phenolic Metabolites During the Reciprocal Gut Microbiome Interactions

**Figure 5:**
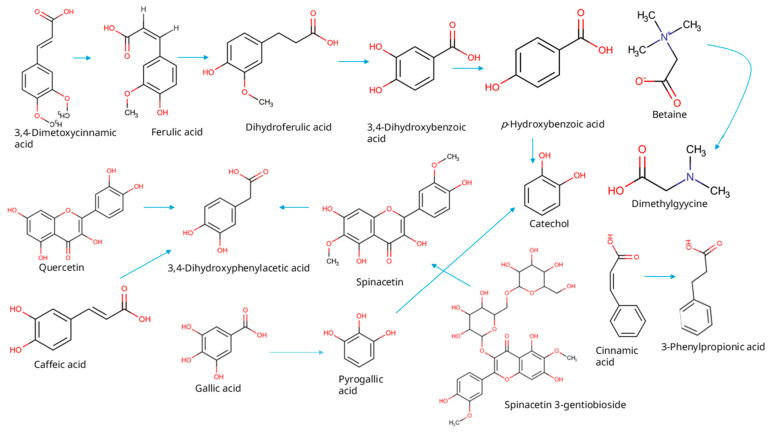
Proposed mechanistic pathways of phenolic biotransformation during reciprocal gut microbiome interactions in baby spinach.

The metabolite profile reflects a coordinated anaerobic aromatic degradation network initiated by glycoside hydrolysis, *O*-demethylation, and reductive side-chain modification. The proposed mechanistic pathways of phenolic biotransformation during reciprocal gut microbiome interactions in baby spinach are given in Figure 5.

Spinacetin 3-gentiobioside and quercetin are first deglycosylated by microbial β-glucosidases predominantly expressed in *Bacteroides* spp. and *Bifidobacterium* spp., releasing aglycones that undergo corrinoid-dependent *O*-demethylation catalyzed by methyltransferase systems reported in *Blautia*, *Eubacterium*, and other *Clostridial Firmicutes* [30,54,55,56,57,58]. Hydroxycinnamic acids (3,4-dimethoxycinnamic acid, ferulic acid, caffeic acid) are reduced at the α,β-unsaturated bond by NADH-dependent hydroxycinnamate reductases (HcrAB-like systems), generating dihydroferulic acid and related intermediates [10,58]. Phenolic acid decarboxylases (PadA-type enzymes) subsequently convert cinnamic acid derivatives into vinyl phenols, while β-oxidation–like side-chain shortening pathways transform cinnamic acid into 3-phenylpropionic acid [10,58]. Flavonol C-ring fission, mediated by quercetinases and related dioxygenase systems characterized in *Flavonifractor plautii* and *Eubacterium ramulus*, yields 3,4-dihydroxyphenylacetic acid [58]. Benzoic acid derivatives (3,4-dihydroxybenzoic acid, *p*-hydroxybenzoic acid) and gallic acid are further processed via reductive dehydroxylation and dearomatization steps involving benzoyl-CoA ligase and benzoyl-CoA reductase–like complexes found in strict anaerobes, generating catechol and pyrogallic acid as central intermediates in anaerobic aromatic metabolism [10,58,59].

These reactions represent an enzymatically regulated carbon channel rather than nonspecific degradation. *O*-demethylation increases substrate reactivity and electron availability; side-chain reduction and decarboxylation decrease redox potential and enhance membrane permeability; and benzoyl-CoA–mediated dearomatization enables ring reduction under anoxic conditions [58]. The progressive formation of catechol-type and phenylpropionate intermediates indicates structured flux through anaerobic aromatic compound degradation pathways conserved in *Ruminococcaceae*, *Peptostreptococcaceae*, and related *Firmicutes* [10,59]. In parallel, betaine undergoes reductive demethylation via glycine betaine reductase and associated methyltransferase systems in certain *Clostridium* spp. and *Proteobacteria*, producing dimethylglycine and contributing to intracellular one-carbon cycling and redox balance during fermentation [60,61]. Collectively, the identified metabolites define discrete nodes within glycoside hydrolase, phenolic acid reductase, phenolic acid decarboxylase, *O*-demethylase, and benzoyl-CoA–dependent pathways, demonstrating that spinach-derived phenolics are systematically transformed through conserved anaerobic enzymatic networks, reflecting functional specialization within the gut microbiome rather than passive chemical breakdown [62]. Together, these metabolomic findings provide mechanistic insight into phenolic transformation during simulated gastrointestinal digestion and subsequent microbial metabolism, demonstrating that both fresh and stored baby spinach undergo structured, enzyme-mediated aromatic conversions. This indicates that phenolic–microbiome interactions remain stable despite storage differences and follow consistent metabolic pathways.

## 4. Conclusions

This study integrated postharvest storage, gastrointestinal digestion, and gut microbial fermentation to evaluate the metabolic fate and functional transformation of baby spinach phenolics. Refrigerated storage (4 °C) better preserved total phenolic content, flavonoid composition, and antioxidant activity, whereas storage at 25 °C accelerated oxidative degradation and compositional changes, particularly during prolonged storage. Simulated gastrointestinal digestion substantially reduced phenolic bioaccessibility, with only a limited proportion of compounds remaining available for colonic fermentation, demonstrating the combined effects of storage and digestion on phytochemical availability. Despite reductions in parent phenolic compounds, in vitro colonic fermentation revealed relatively stable microbial phenolic metabolism and generated a structurally diverse metabolite profile dominated by flavonoid derivatives and phenylpropionic intermediates. Spinach-derived substrates consistently supported key fermentative genera, including *Bacteroides* and *Megasphaera*, while fermentation time exerted a stronger influence on microbial community assembly and metabolite production than storage conditions. These findings suggest that although storage alters the composition of phenolic precursors reaching the colon, gut microbial communities retain the capacity to metabolize diverse substrates into potentially bioactive metabolites.

Nevertheless, this study has several limitations. Fecal inocula were obtained from a small cohort of healthy adult male donors, which may limit generalization to broader populations. In addition, only two storage temperatures and selected storage durations were evaluated, while other postharvest factors such as packaging conditions, light exposure, and humidity were not investigated. The in vitro digestion–fermentation system also represents a simplified model that may not fully capture the complexity of in vivo gastrointestinal and microbiome interactions. Furthermore, the untargeted metabolomics approach provided relative rather than absolute metabolite quantification, and the biological activities of individual gut-derived metabolites were not directly investigated. Future studies should incorporate larger and more diverse donor cohorts, targeted quantitative metabolomics, and advanced dynamic digestion or in vivo models to better understand the bioavailability and physiological relevance of gut-derived phenolic metabolites. Integrating complementary multi-omics approaches, including metagenomics and transcriptomics, may also provide deeper mechanistic insight into food–microbiome interactions and microbial metabolic pathways during digestion and fermentation.

## Data Availability

The original contributions presented in the study are included in the article, further inquiries can be directed to the corresponding author.

## Supplementary Materials

The following supporting information can be downloaded at: https://www.mdpi.com/article/10.3390/foods15111893/s1.
