# Supplementary material for: Metabolomic Characterization of Baby Spinach Phenolics Transformation During Gastrointestinal Digestion and Microbiome-Mediated Metabolism
Source: Foods. 2026 May 27;15(11):1893. doi: 10.3390/foods15111893 (PMC13257288; doi:10.3390/foods15111893)
Supplement: Supplementary file 1 [file foods-15-01893-s001.zip › foods-4296556-supplementary.pdf]

Supplementary Materials Figure S1

Metabolomic Characterization of Baby Spinach Phenolics Transformation during Gastrointestinal Digestion and Microbiome-mediated Metabolism

Akhtar Ali [1], and Leqi Cui [1,*]

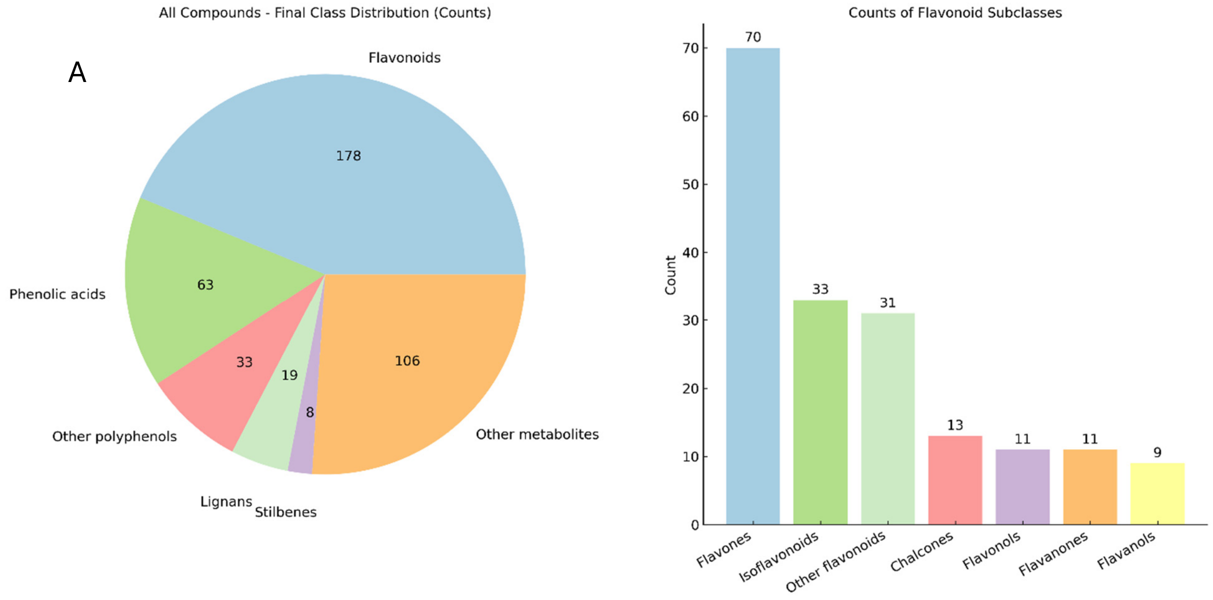

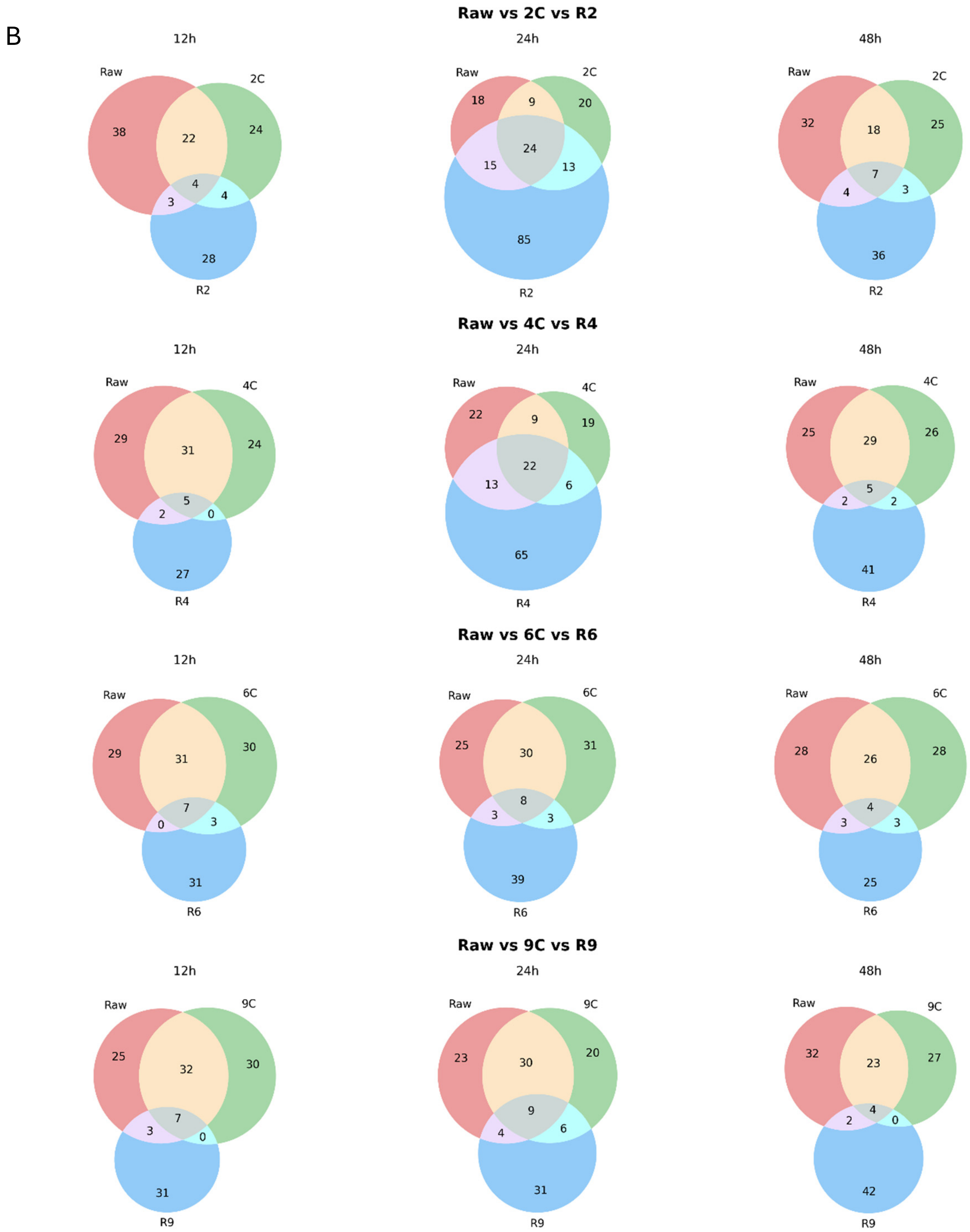
B
Raw vs 2C vs R2
12h
Raw
2C
38
22
24
4
3
4
28
R2
24h
Raw
2C
18
9
20
24
15
13
85
R2
48h
Raw
2C
32
18
25
7
4
3
36
R2
Raw vs 4C vs R4
12h
Raw
4C
29
31
24
5
2
0
27
R4
24h
Raw
4C
22
9
19
22
13
6
65
R4
48h
Raw
4C
25
29
26
5
2
2
41
R4
Raw vs 6C vs R6
12h
Raw
6C
29
31
30
7
0
3
31
R6
24h
Raw
6C
25
30
31
8
3
3
39
R6
48h
Raw
6C
28
26
28
4
3
3
25
R6
Raw vs 9C vs R9
12h
Raw
9C
25
32
30
7
3
0
31
R9
24h
Raw
9C
23
30
20
9
4
6
31
R9
48h
Raw
9C
32
23
27
4
2
0
42
R9

C

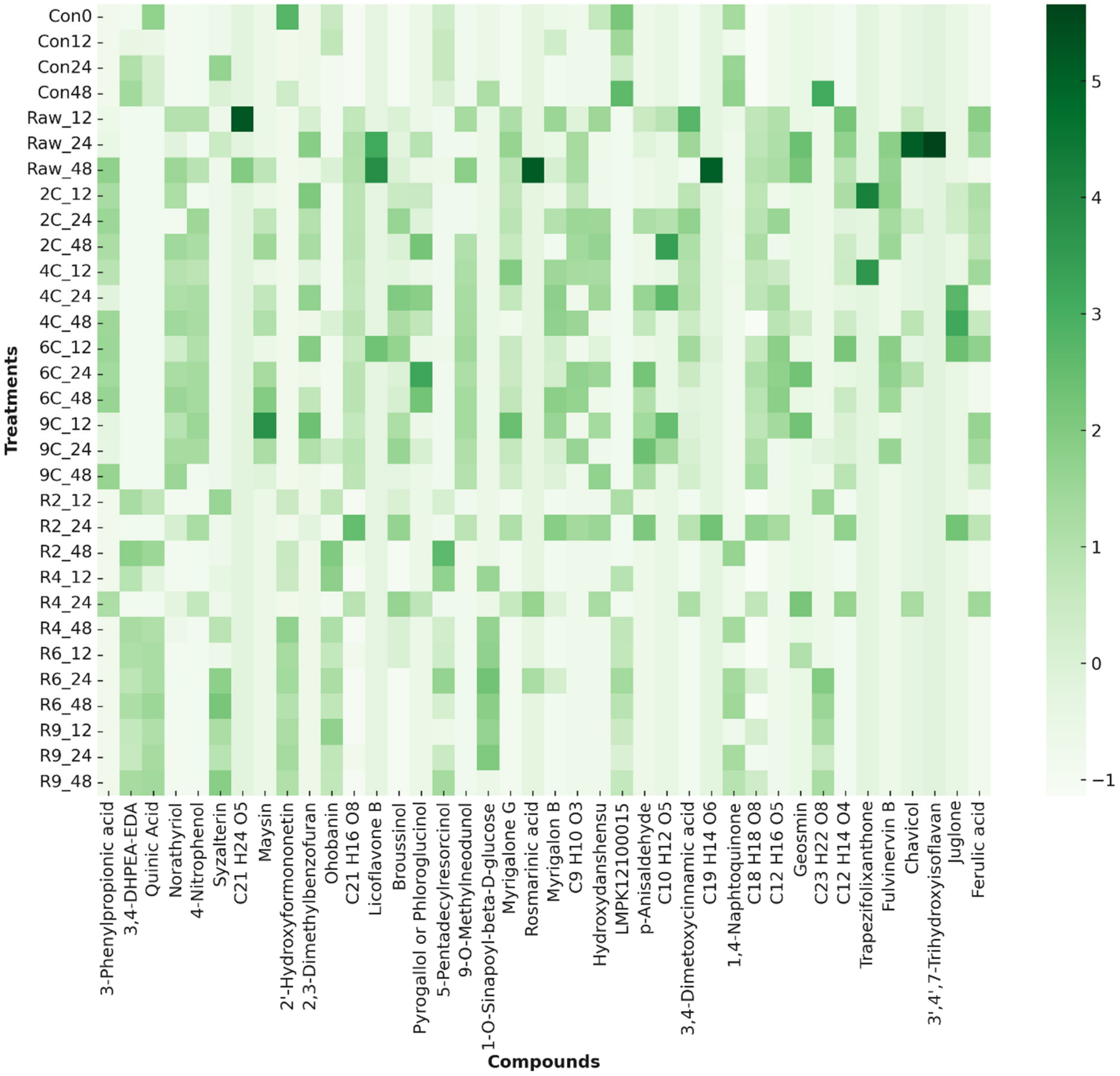
Treatments
Con0
Con12
Con24
Con48
Raw_12
Raw_24
Raw_48
2C_12
2C_24
2C_48
4C_12
4C_24
4C_48
6C_12
6C_24
6C_48
9C_12
9C_24
9C_48
R2_12
R2_24
R2_48
R4_12
R4_24
R4_48
R6_12
R6_24
R6_48
R9_12
R9_24
R9_48
Compounds
3-Phenylpropionic acid
3,4-DHPEA-EDA
Quinic Acid
Norathyriol
4-Nitrophenol
Syzalterin
C21 H24 O5
Maysin
2'-Hydroxyformononetin
2,3-Dimethylbenzofuran
Ohobanin
C21 H16 O8
Licoflavone B
Broussinol
Pyrogallol or Phloroglucinol
5-Pentadecylresorcinol
9-O-Methylneodunol
1-O-Sinapoyl-beta-D-glucose
Myrigalone G
Rosmarinic acid
Myrigalon B
C9 H10 O3
Hydroxydanshensu
LMPK12100015
p-Anisaldehyde
C10 H12 O5
3,4-Dimetoxycinnamic acid
C19 H14 O6
1,4-Naphtoquinone
C18 H18 O8
C12 H16 O5
Geosmin
C23 H22 O8
C12 H14 O4
Trapezifolixanthone
Fulvinervin B
Chavicol
3',4',7-Trihydroxyisoflavan
Juglone
Ferulic acid
5
4
3
2
1
0
−1

D

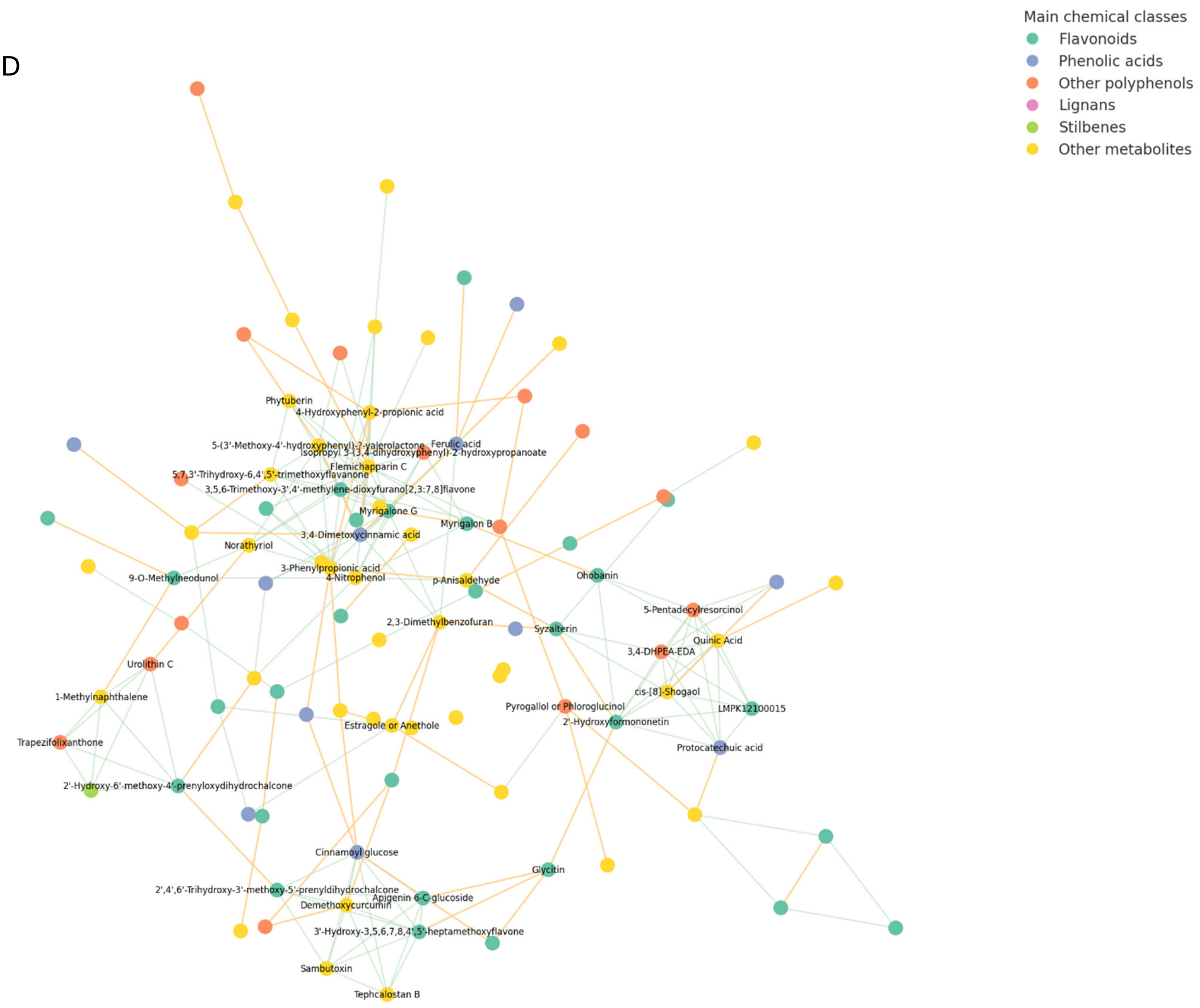

Figure S1. The phenolic metabolites after gut fermentation of raw and stored spinach (4 °C and 25 °C; 2–9 days). (**A**) Chemical class distribution of detected metabolites and relative abundance of flavonoid subclasses. (**B**) Venn diagrams showing shared and unique metabolites across storage conditions in raw and stored spinach. (**C**) Heatmap of selected metabolites across treatments during 12, 24 and 48 h in vitro colonic fermentation. (**D**) Molecular network illustrating structural relationships among metabolite classes. In the Venn diagram, R represents 25 °C, while C represents 4 °C, with spinach stored for 2, 4, 6, and 9 days.
